# GLP-1 agonist, semaglutide use in acute pulmonary embolism recovery: a four-week proof-of-concept study including proteomic profiling

**DOI:** 10.1093/ehjopen/oeaf170

**Published:** 2025-12-24

**Authors:** Chinthaka B Samaranayake, Yen-Cheng Chen, Min Fang, Christopher J Rhodes, Shanshan Song, Farah Sabrin, Ali Ashek, Kathleen Bonnici, Bhashkar Mukherjee, Luke S Howard, Joy Pinguel, Bhavin Rawal, Tom Semple, Laura C Price, S John Wort, Timothy Rudd, Lan Zhao, Colm McCabe

**Affiliations:** National Pulmonary Hypertension Service, Royal Brompton Hospital, part of GSTT Foundation Trust, Sydney Street, London SW3 6NP, UK; National Heart and Lung Institute, Imperial College, London, SW7 2AZ, UK; Analytical and Biological Sciences, Medicines and Healthcare Products Regulatory Agency, Blanche Lane, Potters Bar, Hertfordshire, EN6 3QG, UK; National Heart and Lung Institute, Imperial College, London, SW7 2AZ, UK; National Heart and Lung Institute, Imperial College, London, SW7 2AZ, UK; National Heart and Lung Institute, Imperial College, London, SW7 2AZ, UK; National Heart and Lung Institute, Imperial College, London, SW7 2AZ, UK; Department of Acute Medicine, Chelsea and Westminster Hospital NHS Foundation Trust, 369 Fulham Rd., London SW10 9NH; National Pulmonary Hypertension Service, Royal Brompton Hospital, part of GSTT Foundation Trust, Sydney Street, London SW3 6NP, UK; Department of Respiratory Medicine, St Thomas’ Hospital, Part of GSTT Foundation Trust, Westminster Bridge Road, London SE1 7EH, UK; National Heart and Lung Institute, Imperial College, London, SW7 2AZ, UK; National Pulmonary Hypertension Service, Hammersmith Hospital, Imperial Healthcare NHS Trust, Du Cane Rd, London W12 0HS, UK; National Pulmonary Hypertension Service, Royal Brompton Hospital, part of GSTT Foundation Trust, Sydney Street, London SW3 6NP, UK; National Pulmonary Hypertension Service, Royal Brompton Hospital, part of GSTT Foundation Trust, Sydney Street, London SW3 6NP, UK; National Pulmonary Hypertension Service, Royal Brompton Hospital, part of GSTT Foundation Trust, Sydney Street, London SW3 6NP, UK; National Pulmonary Hypertension Service, Royal Brompton Hospital, part of GSTT Foundation Trust, Sydney Street, London SW3 6NP, UK; National Heart and Lung Institute, Imperial College, London, SW7 2AZ, UK; National Pulmonary Hypertension Service, Royal Brompton Hospital, part of GSTT Foundation Trust, Sydney Street, London SW3 6NP, UK; National Heart and Lung Institute, Imperial College, London, SW7 2AZ, UK; Analytical and Biological Sciences, Medicines and Healthcare Products Regulatory Agency, Blanche Lane, Potters Bar, Hertfordshire, EN6 3QG, UK; National Heart and Lung Institute, Imperial College, London, SW7 2AZ, UK; National Pulmonary Hypertension Service, Royal Brompton Hospital, part of GSTT Foundation Trust, Sydney Street, London SW3 6NP, UK; National Heart and Lung Institute, Imperial College, London, SW7 2AZ, UK

**Keywords:** Acute pulmonary embolism, GLP-1 agonist, Venous thromboembolism, Proteomics, Semaglutide

## Abstract

**Aims:**

Vasorelaxant and anti-inflammatory properties of glucagon-like peptide-1 (GLP-1) agonists support their investigation in aiding the recovery of patients with acute pulmonary embolism (PE) at risk of worse outcomes.

**Methods:**

We undertook a four week non-randomized, controlled open-label study examining proteomic changes, markers of vascular inflammation and exploratory imaging endpoints in response to GLP-1 agonist, semaglutide (0.25 mg weekly) added to standard of care anticoagulation in patients with intermediate high-risk PE.

**Results:**

44 plasma proteins were downregulated in response to semaglutide that were significantly enriched for glycoproteins (false discovery rate q < 0.01). Glycopeptide analysis of highly abundant glycoproteins between diagnosis and follow-up demonstrated a reduction in glycopeptide abundance suggesting protein deglycosylation as a possible mechanism of glycoprotein down-regulation. Down-regulated proteins included regulators of metabolic stress and complement pathway intermediates, which were at higher abundance in PE patients at diagnosis compared to age and sex-matched controls without PE (all *P* < 0.001). Exploratory evaluation of radiological markers of right ventricular dysfunction improved from baseline to follow-up only in patients who received semaglutide (*P* < 0.01).

**Conclusions:**

These findings suggest merit in wider investigation of immunometabolic changes in the plasma proteome during acute PE recovery and their potential relevance to modulation using GLP-1 agonists.

**Registration:**

The study was registered under clinicaltrials.org (NCT06118203).

## Graphical Abstract

**Graphical Abstract:**
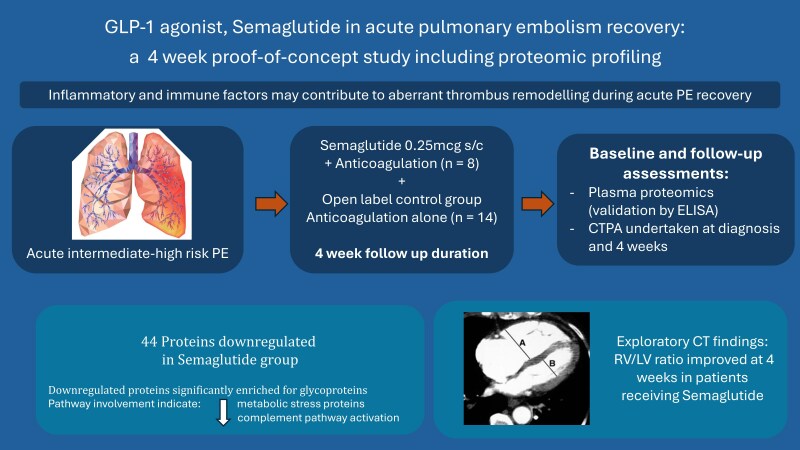

## Introduction

Multiple studies demonstrate that in up to 50% of patients suffering acute pulmonary embolism (PE), lung perfusion recovery at follow-up remains incomplete despite adequate compliance with anticoagulation.^1^ Risk factors for impaired vascular recovery include a higher European Society of Cardiology (ESC) risk category at PE presentation and a larger presenting clot burden.^2^ However, molecular mechanisms that govern aberrant pulmonary thrombus remodelling and impaired recovery in right ventricular function, which in extreme circumstances lead to chronic thromboembolic pulmonary hypertension (CTEPH), remain unknown. Additional management strategies that optimize recovery of both pulmonary blood flow and right ventricular function in patients with higher ESC risk status at PE presentation remain a key objective.

Glucagon-like peptide-1 (GLP-1) agonists carry wide-ranging anti-inflammatory and immunomodulatory effects primarily mediated through the GLP-1 receptor (GLP-1R), which is highly expressed in the heart and lung vasculature.^3^ Reduction in vascular inflammation, oxidative stress, and vasoconstriction have also been demonstrated in multiple vascular beds in response to GLP-1 agonists supporting a potentially beneficial role for their use in acute PE as pulmonary thrombus remodels and injured vessels recanalize. Here, we report an open-label, controlled pilot study evaluating the use of GLP-1 agonist semaglutide administered at 0.25 mg weekly as an add-on therapy to standard of care anticoagulation for 4 weeks in adult patients with acute intermediate–high-risk PE. The study included exploratory effects of semaglutide on the plasma proteome in acute PE as well as on computed tomography (CT)-based metrics of RV dysfunction assessed between diagnosis and follow-up.

## Methods

A total of 22 patients were recruited at four sites in London, UK. All participants underwent sample collection, clinical examination, and CT imaging at two time points: firstly, within 48 h of diagnosis of acute PE (baseline) and secondly, at 4 weeks (follow-up). Adult patients with intermediate–high-risk acute PE initiated on anticoagulation in hospital were eligible. Exclusion criteria included the presence of significant co-existent pulmonary and/or cardiac disease or active malignancy; those currently taking GLP-1 agonists, sulphonylureas, or insulin for diabetes mellitus; and those with standard contraindication to GLP-1 agonist use, e.g. estimated glomerular filtration rate (eGFR) < 30 mL/min. Baseline computed tomography pulmonary angiogram (CTPA) scans were undertaken at presentation in the emergency department. Patients enrolled in the study were given the choice of receiving either standard of care or semaglutide administered in addition to standard of care as determined by the treating clinician. Study investigators were not involved in the allocation of patients into study groups. Additionally, the laboratory analyses and radiological assessments were performed by investigators blinded to patient group allocations. For the proteomics analysis, an unbiased and blinded biostatistical analysis was conducted for each protein using a linear mixed-effects model. Commercially available enzyme-linked immunosorbent assay (ELISA) kits were used for quantification of plasma chemokines (R&D systems, MN, USA) using standard laboratory protocols with targeted proteins validated using a Complement Luminex assay with Bio-plex Manager software. Ethical approval was obtained from the local Human Research Ethics Committee (21/PR/1738). The study was sponsored by Imperial College, London, and registered under clinicaltrials.org (NCT06118203).

## Results

**Figure 1:**
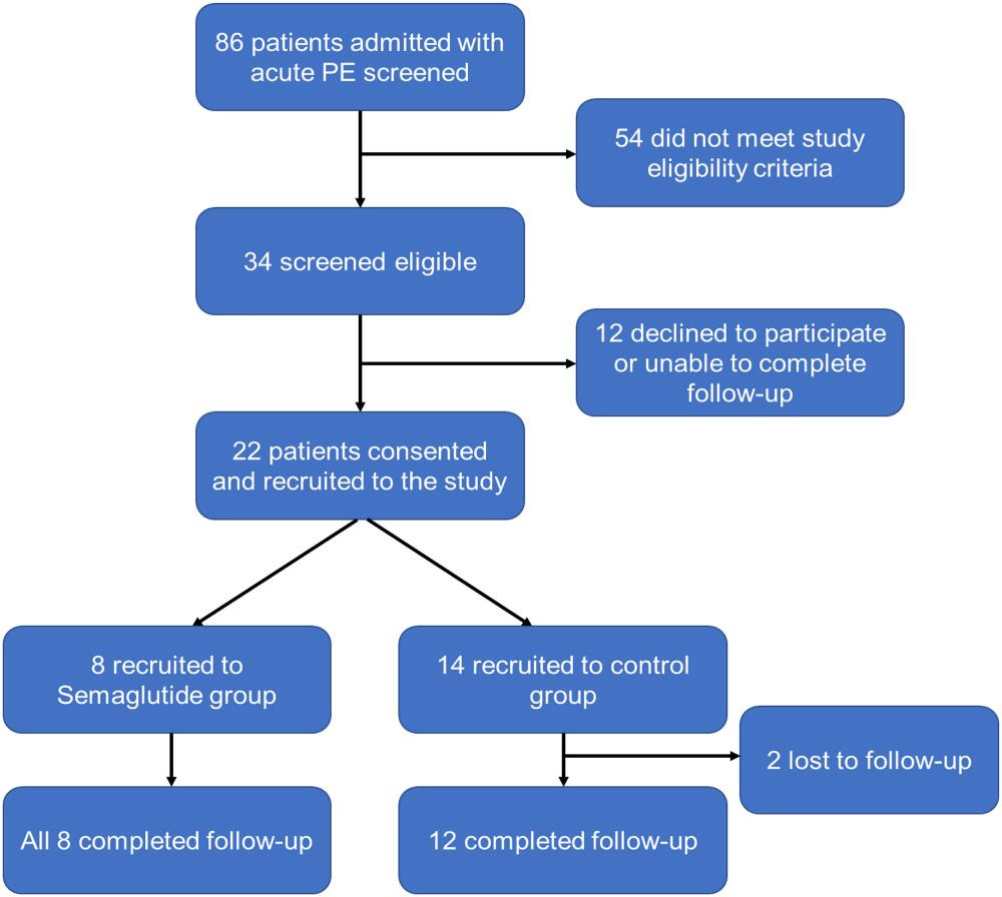
Patient recruitment and study overview.

Twenty-two patients [median age 66 years, interquartile range (IQR) 55.5–72.5] were allocated as follows: semaglutide group (anticoagulation + semaglutide 0.25 mcg s/c weekly, *n* = 8) and control group (anticoagulation alone, *n* = 14) following a 12-month recruitment period (*Figure 1*). Baseline demographics and clinical characteristics are shown in *Table 1*. No tolerability signals were observed with semaglutide during the study. Plasma biomarkers reflecting changes in endothelial function were analysed at baseline and follow-up. Plasma matrix metalloproteinase-9 (MMP-9) level reduced in response to semaglutide [median level 56.5 pg/mL (IQR 29.4) at baseline and 38.6 pg/mL (IQR 25.3) at follow-up, *P* = 0.028] in contrast to controls [median level 56.1 pg/mL (IQR 38.8) at baseline and 46.4 pg/mL (IQR 24.9) at follow-up, *P* = 0.594]. No change was seen in plasma intracellular adhesion molecule-1 (ICAM-1), vascular cell adhesion molecule-1 (VCAM-1), or E-selectin levels during the study in either control or semaglutide groups.

**Table 1:** Patient demographics, radiological characteristics, and treatment allocations divided by group allocation to semaglutide and control arms

Characteristics		Control group *N* = 14	Semaglutide group *N* = 8	*P* value
Demographics				
Age/years (median/ IQR)		59 (49–66)	58 (56–68)	0.23
Gender female (*n*/%)		6 (42.9)3 (37.5)	3 (37.5)	–
Anthropometrics				
BMI kg/m^2^ (mean/SD)		25.5 (5.3)	28.1 (5.7)	0.34
Clinical parameters on presentation				
PESI score (mean/SD)		123.0 (25.7)	136.1 (28.1)	0.33
PESI score	Class V—very high risk (*n*/%)	7 (50.0)	4 (50.0)	0.59
	Class IV—high risk (*n*/%)	1 (7.1)	2 (25.0)	0.25
	Class III—moderate risk (*n*/%)	6 (42.9)	2 (25.0)	0.18
CTPA characteristics				
Most proximal clot location	Saddle (*n*/%)	2 (14.3)	2 (25.0)	0.35
	Main PA (*n*/%)	7 (50.0)	4 (50.0)	0.76
	Lobar arteries (*n*/%)	5 (35.7)	2 (25.0)	0.51
Clot number	Bilateral	14 (100)	8 (100)	–
RV to LV diameter ratio (mean/SD)		1.18 (0.26)	1.40 (0.37)	0.17
Laboratory markers				
Elevated troponin (*n*/%)		14 (100)	8 (100)	–
eGFR/mL/min/1.73 m^2^ (mean/SD)		83.7 (19.8)	78.7 (9.7)	0.43
Initial treatment				
Intravenous heparin infusion (*n*/%)		3 (21.4)	2 (25.0)	0.74
Low molecular weight heparin (*n*/%)		8 (57.1)	6 (75.0)	0.66
Catheter-directed thrombolysis (*n*/%)		3 (21.4)	0 (0)	–
Discharge anticoagulation				
Apixaban (*n*/%)		14 (100)	8 (100)	–

DOAC, direct oral anticoagulant; IQR, interquartile range; SD, standard deviation; BMI, body mass index; PA, pulmonary artery; CTPA, computed tomography pulmonary angiogram; SBP, systolic blood pressure; SpO_2_, oxygen saturation; PESI, pulmonary embolism severity index; LV, left ventricle; RV, right ventricle, RVSP, right ventricular systolic pressure.

**Figure 2:**
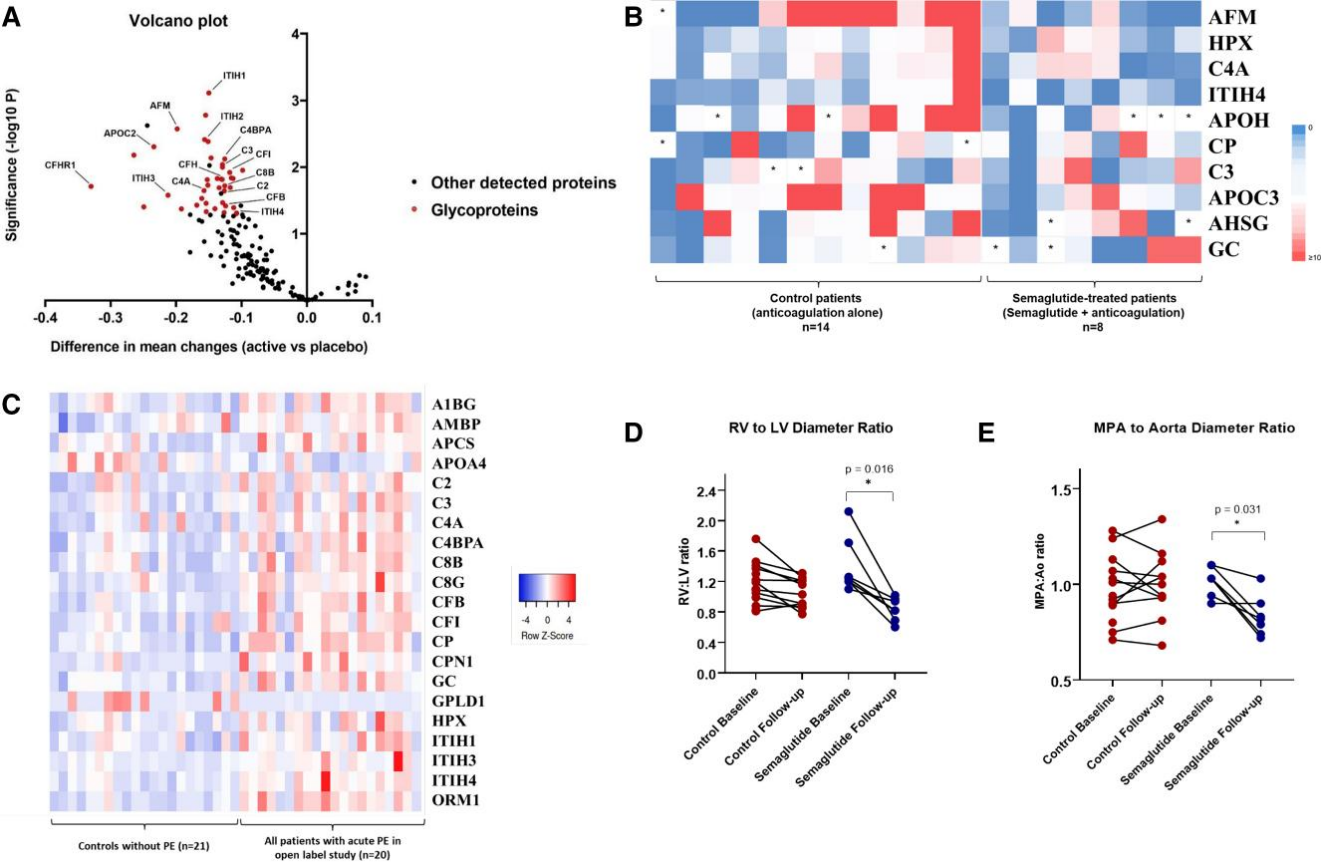
(*A*) Differential protein expression in response to semaglutide treatment added to anticoagulation in acute pulmonary embolism between baseline and follow-up. (*B*) Heatmap of ratios of glycopeptide abundances between (0 to >10×, N-glycopeptides plus O-glycopeptides) baseline and follow-up plasma samples in control (anticoagulation alone)- and semaglutide (semaglutide + anticoagulation)-treated patients (note two missing controls without follow-up samples) * = no glycopeptides identified. (*C*) Quantification of protein abundance in all pulmonary embolism patients sampled at diagnosis (baseline) (*n* = 20, given two samples with insufficient protein identification) compared to contemporaneous age- and sex-matched controls in whom pulmonary embolism had been excluded (*n* = 21). (*D*) Changes in the right ventricle to left ventricle diameter ratio in control and semaglutide groups. (*E*) Change in the main pulmonary artery to aorta diameter ratio in control and semaglutide groups. AFM, afamin; AMBP, alpha-1-microglobulin/bikunin precursor; APCS, amyloid P complement serum; APO A4, apolipoprotein A4 and, C, respectively; A1BG, alpha-1-B glycoprotein; ITIH-1-4, inter-alpha-trypsin inhibitor heavy chain-1-4; CP, ceruloplasmin; CPN1, carboxypeptidase N subunit 1; C2, 3, 4A, 8B, Complement Component 2, 3, 4A, and 8B, respectively; C4BPA, C4b binding protein alpha-chain; CFB, CFH, CFHR-1, CFI, Complement Factor B, H, H-related-1, and I, respectively; GC, GC vitamin D binding protein; GPLD1, GPI-specific phospholipase D1; HPX, haemopexin; ORM1, orosomucoid 1; HPX, haemopexin; APOH, beta-2-glycoprotein 1.

Plasma proteomics included measurement of 2554 circulating proteins. Examining all well-detected proteins in response to semaglutide, a total of 44 proteins were nominally significantly altered during the study (*P* < 0.05), although none individually met significance for multiple testing. Enrichment testing of these 44 proteins identified significant enrichment for glycoproteins (40/44 proteins, false discovery rate q < 0.05). Expression of these glycoproteins showed a clear down-regulation pattern post-treatment (*Figure 2A*) and included multiple complement and metabolic stress mediators, which were further validated by ELISA (data not shown). Ten highly abundant glycoproteins with multiple glycosylation sites were compared across patients receiving semaglutide and those receiving standard of care. Semaglutide-treated patients showed a clear pattern of down-regulation in protein glycosylation compared to the control group (*Figure 2B*).

Given evidence for glycoprotein enrichment and complement pathway down-regulation in response to semaglutide, protein abundances were remeasured at diagnosis across all acute PE patients (*n* = 20) as well as within a second cohort of age- and sex-matched controls, a population undergoing contemporaneous investigation for unexplained breathlessness in whom thromboembolic disease had been excluded (*n* = 21). This showed higher abundance of complement and metabolic stress proteins in patients diagnosed with acute PE (*Figure 2C*).

Computed tomography pulmonary angiogram-derived parameters between baseline and follow-up for each patient demonstrated significant improvements in both the right ventricle (RV) to left ventricle (LV) diameter ratio (*P* = 0.016) and main pulmonary artery (mPA) to aorta (Ao) diameter ratio (*P* = 0.031) in the semaglutide group with no change seen in the control group (*P* > 0.05) (*Figure 2D* and *E*). All patients had a significant reduction in thrombus burden at follow-up compared to baseline assessed by the Qanadli index (*P* = 0.018, controls vs. *P* = 0.003, semaglutide-treated patients).

## Discussion

Here, we present the results of a first-in-disease pilot study evaluating the GLP-1 agonist semaglutide in intermediate–high-risk acute PE patients. Administration of semaglutide, administered within 48 h of diagnosis at 0.25 mg weekly, was well tolerated over a 4-week period and was associated with significant down-regulation of plasma glycoproteins. Glycopeptide analysis of highly abundant glycoproteins across all patients revealed a deglycosylating effect of semaglutide as the mechanism of glycoprotein down-regulation. The increased abundance of complement and metabolic stress glycoproteins observed at the time of PE presentation supports a therapeutic rationale for GLP-1 agonist-mediated immunomodulation in selected patients with acute PE. However, further investigation in larger cohorts is required to understand the functional significance of semaglutide as an add-on treatment in this setting.

These data extend previous findings in patients with acute PE, which support an emerging role of circulating immune and inflammatory factors in PE recovery.^4,5^ MMP-9, down-regulated by semaglutide in our study, has been associated with increased long-term pulmonary vascular wall fibrosis in animal thrombosis models, suggesting potential merit in its down-regulation during the recovery phase of PE.^6,7^ Ficolin 3, Complement Factor B, and Factor H have also been implicated in the progression of vascular inflammation and coagulation homeostasis and were down-regulated in semaglutide-treated patients.^4,5,8,9^ In addition to immune factors, highly glycosylated coagulation and platelet surface membrane proteins responsible for maintenance of haemostatic balance as well as glycosylation-related enzyme activity that varies by vascular bed of origin and body mass index (BMI) support potentially novel mechanisms determining the natural history of PE recovery, which may be potentially influenced by semaglutide.^10^ Evaluation of protein glycosylation trends in acute PE between diagnosis and recovery requires further investigation to better contextualize our findings. In addition, future administration of semaglutide potentially at different doses and durations are required to understand the therapeutic potential of modulating vascular inflammation during acute PE recovery.

Limitations in our study include its restriction to patients in whom in-hospital treatment was mandated with an unequal distribution of disease severity and adjunctive therapies between groups that included catheter-directed thrombolysis in three control patients. These factors are intrinsic to a small, non-randomized study, which was also underpowered to detect clinical effects of semaglutide, meaning radiological endpoints should only be regarded as hypothesis generating. Here, although a reduction in RV strain was observed in semaglutide-treated patients, the design of our study, which left treatment allocation at the discretion of the patient, may have introduced selection bias with better compliant patients choosing an experimental therapy out of curiosity. However, effort was made via provision for weekly phone calls to maximize treatment compliance, which included verification of anticoagulation compliance across both groups. In conclusion, current PE treatment guidelines in intermediate–high-risk PE present limited evidence for long-term recovery via use of acute reperfusion strategies given the frequently unacceptably high risk of haemorrhagic complication. Our findings suggest merit in wider investigation of the relevance of immunometabolic changes in the plasma proteome during PE recovery and their potential modulation using GLP-1 agonists.

## Data Availability

The data underlying this article are available in the article. No authors have any conflicts of interest with respect to the content of this article.
